# OveRcoming Adverse ChiLdhood Experiences (ORACLE): A Mixed Methods Intervention Co-design Study to Improve Outcomes for Children and Young People Experiencing or at Risk of Adversity

**DOI:** 10.1007/s10935-025-00866-7

**Published:** 2025-08-11

**Authors:** Julia R. Forman, Ruth McGovern, Sophie G. E. Kedzior, Harriet Boulding, Simon Barrett, Cassey Muir, Nicholas Kofi Adjei, Yoko V. Laurence, Tianne Haggar, Julia Fox-Rushby, David Taylor-Robinson, Eileen Kaner, Ingrid Wolfe

**Affiliations:** 1 Department of Women and Children’s Health, School of Life Course and Population Sciences, King’s College London, London, UK; 2 Population Health Sciences Institute, Newcastle University, Newcastle upon Tyne, UK; 3 The Policy Institute, King’s College London, London, UK; 4 Department of Public Health, Policy and Systems, University of Liverpool, Liverpool, UK; 5 Department of Population Health Sciences, School of Life Course and Population Sciences, King’s College London, London, UK

**Keywords:** Intervention design, Mixed methods, Co-design, Adverse childhood experiences, Mental health, Substance misuse, Domestic abuse, Syndemics

## Abstract

Childhood exposures to adversity are common and increase risk for negative health and social outcomes throughout the life course. There is limited evidence regarding interventions to prevent or reduce the impact of adverse childhood experiences (ACEs), particularly for families with multiple adversities. Here we present the findings of mixed methods research to co-design a complex intervention to prevent adverse childhood experiences, and their impacts. Using established research methods, and the framework of the Medical Research Council (MRC) complex interventions development guidance, the work was conducted in four stages, shaped by stakeholder engagement and input at every stage. The first stage, Discover, was exploratory and employed evidence synthesis and quantitative (n = 11,564) and qualitative (n = 31) research methods to understand needs, experiences, and evidence gaps. The Define stage developed three intervention principles and identified intervention options, through a series of six co-design workshops with 41 participants and an academic research team workshop. The Develop and Deliver stages were undertaken through a Policy Lab (22 participants), and developed options for intervention design, before converging on a defined intervention that could be delivered and tested. Through this process, we developed a ‘village-style’ intervention, which functions at three levels: individual service users, operational, and system/strategy. Central to this are link or community health workers who would build relationships with family members, and act as a single point of contact. They should develop an understanding of family needs and the interaction of multiple complex adversities, and advocate for families, facilitating access to services. Crucially, they should use this understanding to work at and feed into operational and strategic levels to reshape services and enhance access for all families at risk of or experiencing adversity. Entry into the intervention through assessments at existing universal touch points, for example at routine perinatal or newborn appointments, should provide a prevention focus and follow the principles of proportionate universalism. Sensitive enquiry regarding financial stress may be a component of the assessment, in response to the findings of this work regarding the contexts created through the interaction of poverty and other adversities. The proposed intervention is designed to improve individual and family outcomes, and generate positive system-level changes. A feasibility study and evaluation will be required in future work, to assess the effects, costs and benefits. The processes and frameworks we developed and used may provide an adaptable template for future intervention co-design work.

**Supplementary Information:**

The online version contains supplementary material available at 10.1007/s10935-025-00866-7.

## Introduction

An estimated 5,410,000 (45.9%) children aged 0–17 in the UK grow up in households with an adult affected by substance use, mental health problems, or domestic violence. For 2,150,000 (18.2%) children, these problems are severe, meaning they are exposed to drug or alcohol dependency, severe symptoms of mental disorders, or parental domestic violence within the last year (Chowdry, 2018). It is estimated that 3.8%–14.9% of UK children experience two of these adversities and 0.24%–3.6% experience all three (Adjei et al., 2022a; Chowdry, 2018). In the US, 27.0% of young adults (aged 18–24) report childhood exposure to household mental illness, 16.9% to witnessing intimate partner violence, and 27.5% to household substance use (Swedo, 2023). In Europe, among young adults (18–25), 11.4% report childhood exposure to household mental illness, 12.9% to caregiver intimate partner violence, 16.0% to household alcohol abuse, and 2.8% to household drug abuse (Bellis et al., 2023).

Early life experiences significantly impact health throughout the life course (Britto et al., 2017; Marmot et al., 2010). Early adverse experiences can have immediate and long-lasting impacts (Bellis et al., 2015), and major health and financial burdens globally (Hughes et al., 2021). Children exposed to adversity are more likely to experience ill-health (Artz et al., 2014), injuries (Baker et al., 2015), cognitive and language development delays (Bagur et al., 2022; Barnard, 2006), lower educational attainment (Berg et al., 2016; Cleaver et al., 2011) and behavioural problems (Evans et al., 2008; Grip et al., 2012; McGovern et al., 2023). Children whose parents use substances are more likely to use substances (McGovern et al., 2018) and children exposed to domestic violence are more likely to be involved in domestic violence in adulthood (Murrell et al., 2007). Multiple adverse experiences further increase the risk of poor health outcomes, including cancer, heart and respiratory disease, problematic drug and alcohol use, mental ill health, and interpersonal and self-directed violence (Hughes et al., 2017). Public health responses to adverse childhood experiences include preventing exposure (e.g. with targeted parenting interventions), intervening with high-risk groups (e.g. specialist domestic violence support), and providing treatment (e.g. for parental mental health and/or substance misuse) (Klevens & Alexander, 2019).

The theory of syndemics suggests that the combined impact of multiple adversities due to harmful social conditions, including poverty, is amplified by their interaction (Tsai et al., 2017). However, evidence regarding the interrelationships between adverse exposures is limited, and few studies consider the lived experience and support needs of affected families. There is a paucity of interventions for populations experiencing clustering of parental substance use, mental health problems and domestic violence (Allen et al., 2022; Barrett et al., 2023), and siloed intervention approaches may not be adequate for multiple interacting and synergistic adversities (Isobe et al., 2020). For populations experiencing multiple risk factors, including parental substance use, mental health problems, and domestic violence, these risk factors may be or create barriers to accessing existing services, particularly in the context of poverty. Interventions co-designed by people with lived experience of multiple adversities may be able to reduce or remove these barriers.

We present the findings of our mixed methods research which set out to co-design a complex intervention to improve outcomes for children and young people experiencing or at risk of adverse childhood experiences, through prevention and improved support.

## Methods

### Frameworks

**Fig. 1:**
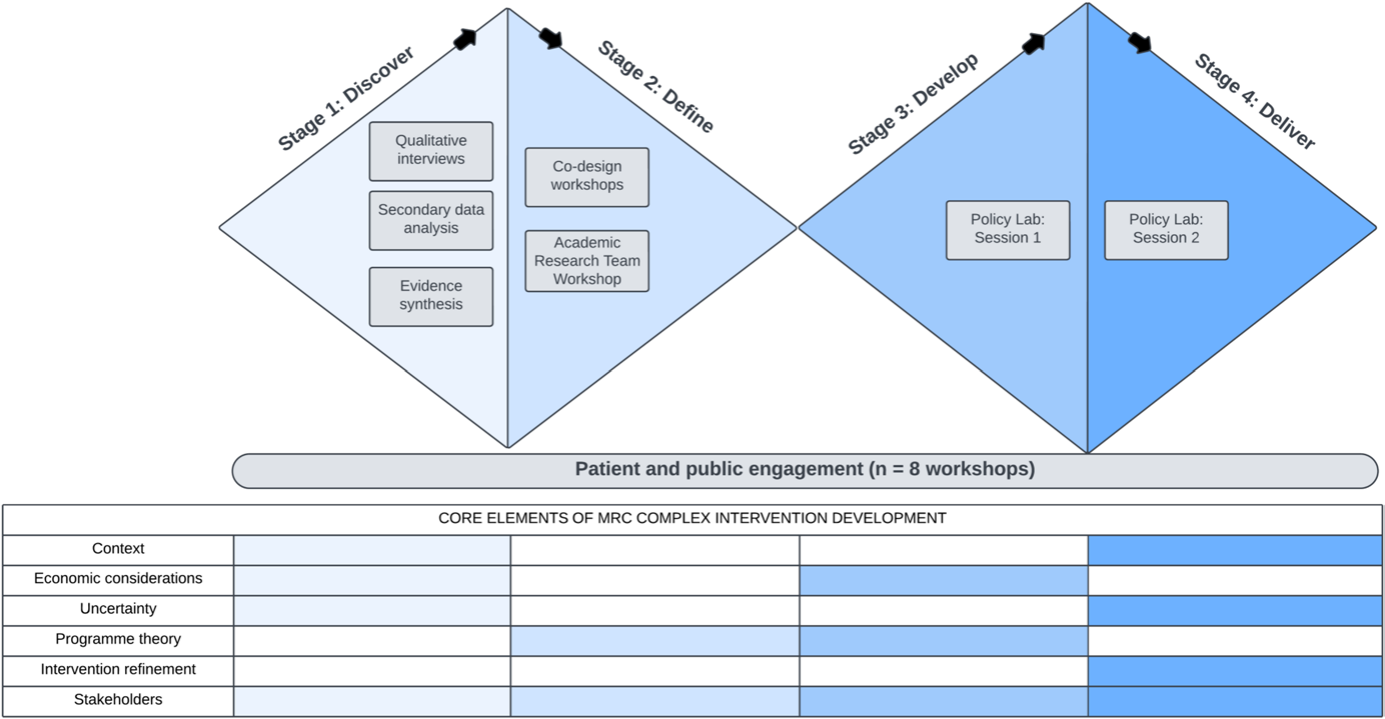
The ORACLE study co-design template, an intervention development process using the Double Diamond model and the MRC framework for complex interventions. The figure illustrates the methods used (grey boxes) in each of the four stages of the Double Diamond process, and the core elements of the MRC complex intervention development framework addressed in each stage

The intervention design was guided by the Medical Research Council (MRC) framework for complex interventions, covering core elements from intervention development to implementation (Skivington et al., 2021). The Double Diamond conceptual framework (British Design Council, 2021) outlines four sequential stages (Discover, Define, Develop, and Deliver) of information gathering and refinement, iterating divergent explorative stages and convergent focusing stages. Figure 1 illustrates the intervention design stages, corresponding work packages, and the MRC Complex Interventions Framework elements addressed in each Stage.

### Stage 1: Discover

The Discover stage aimed to understand needs, experiences, and evidence gaps, using qualitative interviews, longitudinal cohort quantitative analyses, and evidence synthesis. The methods for these work packages are summarised below and where full methods have been reported separately, the references are provided for additional detail.

#### Qualitative Interviews

To understand needs and experiences, semi-structured interviews were conducted with parents (18 mothers, 6 fathers) and young people (4 males, 3 females, ages 14–20) with lived experience of parental mental health problems, substance use, and/or domestic violence and abuse (Kedzior et al., 2024). Eligibility criteria included having at least one of the risk exposures and being in a situation where participating in research would be feasible and appropriate (e.g., families not in immediate crisis). Participants were recruited through community services across England, including charities and support services for these parental risk factors, and interviewed March–October 2022. Participants received a £10 e-voucher for their involvement. The interviews were transcribed, and reflexive thematic analysis was applied to the transcripts (Braun & Clarke, 2006). Codes were generated through an inductive approach. The transcript data was uploaded to spreadsheets (one for parent data, one for young people data), which the researchers used to code the data and gain an overview of the dominant codes. From the parent interviews, initial themes were generated by examining the coded data and discussing with the research team. Codes were assigned to each generated theme, and the themes and subthemes were further refined through an iterative process. The interviews with young people were coded, and the codes were mapped to the same themes and subthemes as the parent data.

#### Longitudinal Cohort Quantitative Analyses

We analysed data from the nationally representative UK Millennium Cohort Study to assess the clustering of trajectories of child poverty and family adversities and their impacts on child behaviour and health outcomes in adolescence. Longitudinal data on 11,564 were used to construct trajectories of poverty, parental mental health, domestic violence and abuse, and alcohol use from age 9 months to 14 years. We assessed the associations with mental and physical health outcomes at ages 14 (Adjei et al., 2022a) and 17 (Adjei et al., 2024). Population-attributable fractions quantified the contribution of mental health problems and poverty to adverse outcomes (Adjei et al., 2022b, 2024).

#### Evidence Synthesis

A systematic review of reviews provided an evidence and gap assessment of the range and effectiveness of interventions for children and families with parental risk factors (Barrett et al., 2023).

A grey literature synthesis included evidence from regional reports, research institutions, and service providers on current UK approaches to prevent or mitigate the impact of ACEs on child outcomes.

A health economics scoping review conducted in PubMed using the terms ‘cost’, ‘cost-effectiveness’ and ‘adverse childhood experiences’ resulted in 199 papers. Titles were reviewed for inclusion, and abstracts reviewed if there was uncertainty. Intervention costs were inflated to 2023 country specific currency values (International Monetary Fund, 2024) and converted to USD using 2023 midyear exchange rates (xe.com, 2024).

### Stage 2: Define

The Define stage aimed to use insights from the Discovery phase to develop intervention principles and identify promising intervention options, through co-design workshops and an academic research team workshop.

#### Co-design Workshops

Researchers identified pre-established peer groups from relevant community-based services. Services were selected to ensure involvement from traditionally under-represented groups, including ethnic minorities, those with care experience, and the LGBTQ + community. Researchers organised workshops in collaboration with these gatekeeper service organisations. Six co-production workshops (each lasting 90–120 min) with mothers (n = 24), fathers (n = 6), and young people (n = 11, aged 13–18, mixed gender including non-binary/trans participants) who experienced multiple adversities were conducted in London and North-East England, September–November 2022. Workshop content and activities were informed by public advisory groups and the findings of the previous stage; the findings about lived experiences and impacts were used to develop scenarios, and the evidence synthesis work provided the starting point for discussions of potential interventions. Data from facilitator notes and activities were analysed thematically, resulting in intervention principles (Muir et al., 2024). Participants received vouchers (£10-£25) for their involvement, in line with national standards for public involvement and engagement.

#### Academic Research Team Workshop

The research team held a two-day workshop to review and synthesize findings, and prioritise intervention types for development. The workshop included input from the National Children’s Bureau, the project’s PPI collaborator organisation (described below in the Stakeholder section), and a practitioner from a link-worker intervention, as interest in link workers had developed through the Stage 1 findings and co-design workshops in Stage 2. The workshop culminated in production of a draft logic model for a novel intervention.

### Stages 3 and 4: Develop and Deliver

The Develop and Deliver stages were the second iteration of divergent and convergent design processes. The Develop stage aimed to develop intervention options, while the final stage aimed to produce a defined intervention for delivery and testing. These stages were conducted in a Policy Lab.

#### Policy Lab

The Policy Institute at King’s College London developed a ‘Policy Lab’ workshop to connect research evidence, policy, and practice changes (Hinrichs-Krapels et al., 2020). Each Policy Lab involves collaborative sessions uniting research, policy, practitioner expertise, and lived experience to assess evidence, understand barriers and constraints to change, and then inform policy options to improve outcomes.

The ORACLE Policy Lab included local and national policymakers (n = 9), academics (8), clinicians (3), and representatives from children’s charities (2) (full details available in the Policy Lab report: https://www.kcl.ac.uk/research/overcoming-adverse-childhood-experiences-oracle-policy-lab-report). Participants received evidence briefing materials before the lab, summarizing the initial ORACLE project findings. The lab comprised two interactive sessions over a full day to address key uncertainties from the previous intervention design phases. A mixture of individual reflection, small group deliberation, and plenary discussion facilitated sharing a range of views. All discussions and physical data (e.g., diagrams and participant notes) were analysed thematically.

*Lab Session 1: Develop*. The first session aimed to broaden thinking and exchange perspectives. The views and voices of families living with adversity were included through a video developed with the Family Research Advisory Group (described in the “Stakeholder engagement and participation” section below).

*Lab Session 2: Deliver*. The afternoon session brought different solutions into focus, with concentration on practical questions around feasible and impactful intervention delivery.

### Crosscutting: Stakeholder Engagement and Participation

As described above and published elsewhere, participants with lived experience and other stakeholders were extensively involved through the qualitative interviews (Kedzior et al., 2024), the co-design workshops (Muir et al., 2024), and the Policy Lab (https://www.kcl.ac.uk/research/overcoming-adverse-childhood-experiences-oracle-policy-lab-report).

In addition, patient and public involvement (PPI) shaped the research throughout all four stages of intervention development. This was in collaboration with members of the National Children’s Bureau’s Family Research Advisory Group (parents) and Young Research Advisors (aged 10–25), facilitated by National Children’s Bureau staff. The Family Research Advisory Group participated in three research advisory workshops, attended by 6–12 parents, providing input on research findings and suggestions for future research and dissemination. They also shared their views in a short film, discussing service provision, tailored support needs, service barriers and shortcomings, and examples of good support. The Young Research Advisors participated in 5 research advisory workshops, attended by 10–15 young people, offering their insights on research findings and methodologies. The research advisory workshops were separate from the co-design workshops described in Phase 2. The research advisory workshops with parents were discussion-based, while those with young people involved interactive activities like brainstorming and scenarios (Leask et al., 2019).

## Results

Figure 1 illustrates the core elements of the MRC complex intervention framework which were addressed in each stage.

### Stage 1: Discover

In this section, we report a synthesis of how the findings of the qualitative interviews (Kedzior et al., 2024), longitudinal cohort quantitative analyses (Adjei et al., 2022a, 2022b), and evidence synthesis (Barrett et al., 2023), taken together, inform intervention design. The results of these Stage 1 methods addressed three core elements of the MRC complex intervention development framework: Context, Uncertainty, and Economic considerations.

#### Context

In the analysis of the qualitative data from the semi-structured interviews, four main themes were identified, and six sub-themes (Kedzior et al., 2024) (Supplementary Materials Table 1). Three of these themes, and their subthemes, were relevant to informing the intervention context, in combination with findings from the other Stage 1 methods.

The first theme, *Cumulative adversity*, captured the difficulties that parents and young people face due to, and exacerbated by, low socioeconomic status. Parents described the impact of poverty on their parenting capacity, and how this exacerbated the harm related to their risk factor(s). For example, some mothers reported that financial pressures prevented them from accessing safe alternative accommodation when living with violent or substance using partners (Kedzior et al., 2024). The secondary analysis of the Millenium Cohort study identified a significant cluster of children exposed to persistent poor parental mental health and poverty (11.1% of the cohort), and this cluster experienced worse outcomes at age 14, particularly socioemotional behavioural problems (Adjei et al., 2022a).

In qualitative interviews, multiple interacting risk factors within a family unit were common. This was described in the second theme, *The impact of syndemic risk*, and its subtheme, *Behavioural impacts*. For example, domestic abuse and substance use were reported to impact on mental health, and victims of domestic abuse sometimes described using substances to cope with their experiences.

This theme also captured parents’ feelings of isolation and stigmatisation, in its second subtheme *Emotional impact and social isolation*. For example, parents described low social support from their extended family and friends, particularly where these relationships had broken down. They also felt disconnected from local communities due to stigma around their mental health, substance use, and/or domestic violence and abuse. Parents reported that their children may have suffered from isolation due to other families not wanting their children to interact with families experiencing these issues. Where families relocated following experiences of domestic violence and abuse, parents also described difficulties “*making friends and then starting again*.”

The parents and young people interviewed emphasized the need for support that addresses the complex needs of each family member. This was captured in the final theme, *Family support*, and particularly the subtheme *Ineffective support*. One father described “*fall[ing] in the cracks*” when mental health services were not offered due to substance misuse, while simultaneously drug treatment services were withheld due to his mental health struggles (Kedzior et al., 2024). However, the systematic review demonstrated a lack of system thinking or structural approaches, with most interventions addressing risk factors in isolation (Barrett et al., 2023). Furthermore, interventions typically targeted individuals, in particular mothers, with fathers seldom considered. Future interventions may need to take an integrated approach, addressing multiple risk factors and including all family members.

The final subtheme *Characteristics of good support*, captured the descriptions by parents and young people interviewed of the key characteristics of good support, particularly in light of the stigma they experienced around parental mental health, substance use, and domestic violence. Parents highlighted that social connections and peer support from others with similar experiences can provide both emotional and practical support, reducing feelings of stigmatisation and isolation and providing information about accessing formal support services. Parents and young people also advocated for interventions delivered by knowledgeable, non-judgemental and trauma-informed practitioners. To implement non-judgemental support, they suggested that interventions should take strengths-based approaches, with a focus on capabilities, successes, and progress. As an example, some parents shared their positive experiences of strengths-based parenting classes; these classes had highlighted what they were doing well and improved their self-confidence, in contrast with services focused on their risk factors or negative circumstances. Parents also emphasized the value of sustained contact with a practitioner, over an extended period, to build trusting relationships (Kedzior et al., 2024).

#### Uncertainty

In Stage 1, three key areas of uncertainty were identified. First, the underlying mechanisms or pathways through which poverty and family adversity interact and affect child outcomes need consideration. Any assumptions about these mechanisms must be considered with regards to the intervention design and programme theory.

The second key uncertainty is how to provide appropriate flexibility in interventions for families with complex needs. Both the qualitative interviews (Kedzior et al., 2024) and systematic review (Barrett et al., 2023) raised questions about how to work with these families, and whether support for children should be alongside or separate from parents. Both parents and young people recognized that family members indirectly exposed to risk factor(s), for example living with a partner or parent who is directly experiencing or exposed to a risk factor, require targeted support as well.

**Fig. 2:**
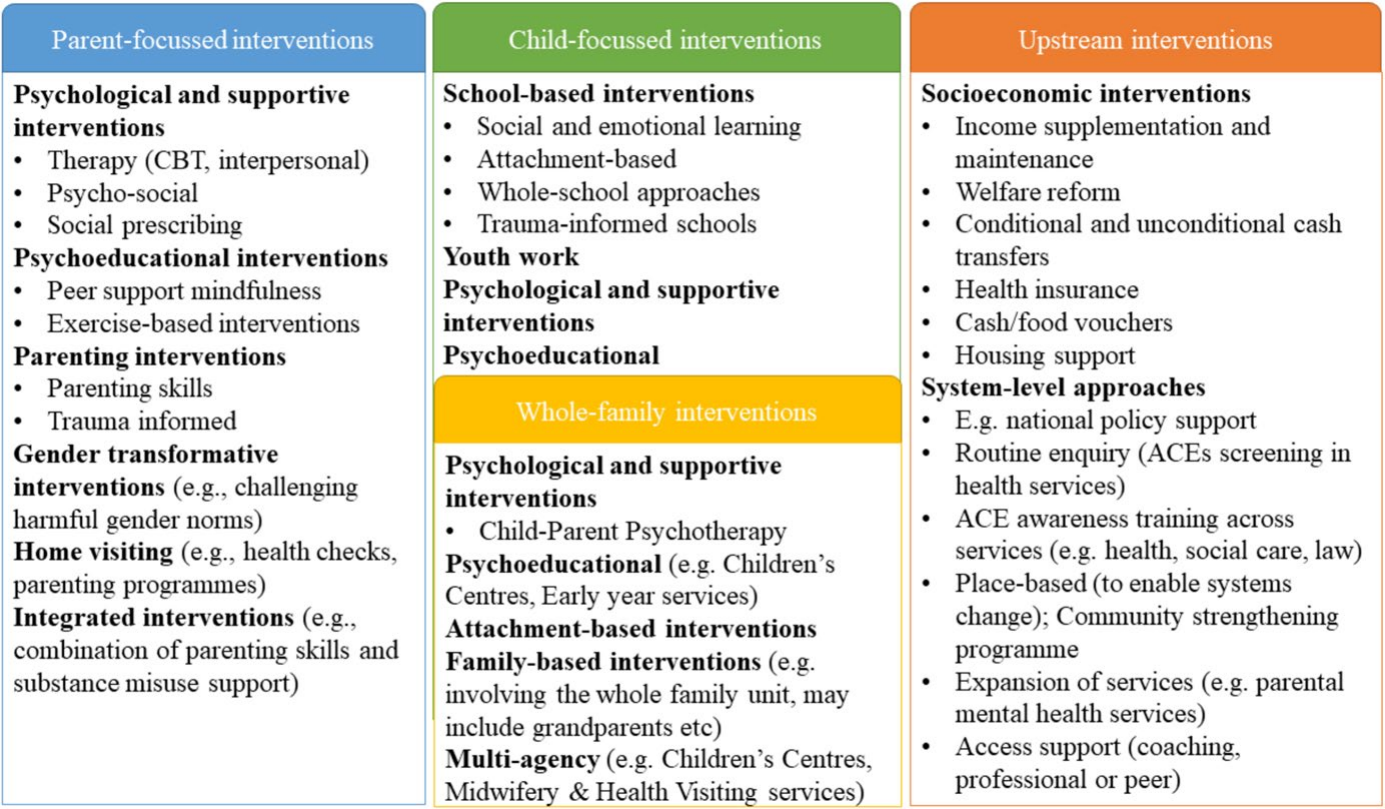
Interventions identified through grey literature and systematic review search, available in the UK for families with parental mental health problems, substance use, domestic violence and abuse, and other ACEs

The third uncertainty is prioritisation and acceptability of intervention approaches. The systematic (Barrett et al., 2023) and grey literature reviews identified myriad intervention approaches including parent-focused, child-focused, whole-family, and upstream (Fig. 2). Evidence for these interventions is variable, partly due to the different intervention components and evaluation strategies employed. The acceptability of these approaches from the perspectives of families and service providers remains uncertain.

These three uncertainties were explored in Stage 2.

#### Economic Considerations

Across the 10 papers identified in the health economics scoping review, costs associated with ACEs varied, with ‘attributable’ costs (a value assigned to ‘lost productivity’ linked to disability adjusted life years attributed to ACEs) or costs of consequences most commonly reported. Annual ACE-attributable costs were estimated for 28 European countries and ranged from US $0.1 billion (Montenegro) to US $129.4 billion (Germany), equivalent to between 1.1% and 6.0% of gross domestic products (Hughes et al., 2021).

Only one study reported intervention costs, which was for the provision of care and support around substance use provided by family members, and this was valued at US $8,863 USD for costs incurred by family members per year in the UK, while US $3,543 was the estimated annual income lost by the carer of the drug using relative (Copello et al., 2010).

### Stage 2: Define

#### Programme Theory

The co-design workshops investigated how an intervention could create positive change, using insights from mothers, fathers, and young people experiencing parental substance use, mental health, domestic violence, and/or poverty. Through the co-design workshops, we identified three underlying intervention principles (Muir et al., 2024).

First, to reduce isolation and loneliness caused by stigma (identified in Stage 1), participating families wanted to build a network to access services and resources. They suggested that this network could include peer support and a dedicated, knowledgeable practitioner. The workshop participants recognised that there were services available locally, noting that, “*Support is out there but we don’t know about it*.” Participants felt peer support networks would help with “*navigation of the support system*”, and wanted a practitioner who could act as a “*family friend”* and “*walk alongside*” them (Muir et al., 2024).

This led the team to invite a practitioner from the “It Takes a Village” program (Kuchemann et al., 2021) to present their model at the Academic Research Team workshop. A key element of the “It Takes a Village” model is the role of practitioners across intervention levels (Supplementary Materials Fig. 1). Practitioners identify barriers to service access through their work listening to families. Then, at the operational level they influence local systems and services to remove barriers, for example through reshaping service protocols and practices, facilitating access and improving the system, not only for families they directly work with, but for all service users.

The second principle developed through the co-design workshops was that to reduce stigma, families “*need to feel understood*” by practitioners. The workshop participants wanted to build relationships with practitioners over extended time periods and in informal settings (i.e., outside healthcare appointments), allowing for opportunities to “*break the ice*”. They also suggested that practitioners be trained in trauma experienced by families facing multiple adversities. Parent participants shared concerns about disclosing family adversity and support needs, often due to fear of child protection services involvement, including the risk of involuntary family separation. Building trusted relationships with practitioners was seen as key to overcoming these concerns, and accessing appropriate support (Muir et al., 2024).

Third, the workshop participants affirmed that all family members’ needs should be considered, an extension of the findings of Stage 1, with “*support for each family member*” and a greater focus on children’s and fathers’ needs. Participants felt that support should be tailored to each family member’s strengths and financial situation, echoing the Stage 1 findings on the interaction of poverty and other adversities.

**Fig. 3:**
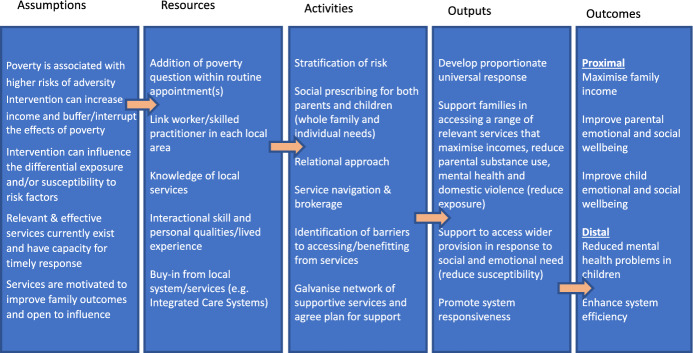
Logic model developed in the Academic Research Team Workshop at the culmination of Stage 2: Define.

Stages 1 and 2 culminated with the production of an initial logic model for a novel ‘village-style’ intervention, outlining assumptions, resources, activities, and the expected outputs and outcomes (Fig. 3). In this model, support to reduce exposures and susceptibility (e.g. poverty reduction by reviewing entitlements to welfare benefits and ensuring families receive all available financial support) should lead to higher incomes and improved parental and child wellbeing, and subsequently improved child mental health, while promoting system responsiveness should enhance system efficiency.

### Stage 3: Develop

The key uncertainties at the culmination of the Define stage were shaped into an overarching question for the Policy Lab in Stages 3 and 4: ‘How could a village-style intervention best be designed to promote early intervention, and deliver positive outcomes for children in families living with complex adversity?’

In the Develop stage, Policy Lab participants built on previous findings, exploring village-style intervention principles and considering different intervention designs, adding to the programme theory and economic considerations.

#### Programme Theory

Policy Lab participants strongly endorsed a village-style approach, emphasizing personalization and coordination of services. They noted numerous barriers to service access, and agreed that a single point of contact (link worker) would best understand the needs of families, and the interaction of multiple complex adversities. Link workers would be advocates, coordinate service access, and prevent families from ‘falling through the cracks’, potentially improving children’s wellbeing, family relationships, school attendance, and social and economic opportunities.

Participants also noted the potential benefits for local services and systems, including that staff may experience increased personal and professional satisfaction through improving outcomes for families, enhancing system integration, multi-sectoral collaboration, and upskilling.

#### Economic Considerations

Participants felt the village-style approach may offer a good return on investment, by improving family outcomes and system costs, in the short- and long-term. However, concerns remained about the feasibility of an initial financial outlay without guaranteed short-term returns.

### Stage 4: Deliver

**Fig. 4:**
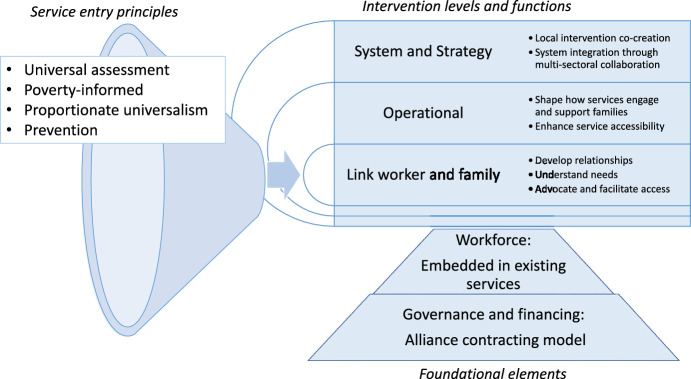
The resulting intervention design can be described in three domains: (1) Service entry principles. Entry into the ‘Village’ would be through assessments at existing universal touch points, for example at routine perinatal or health visiting appointments, providing a prevention focus and following the principles of proportionate universalism. Sensitive enquiry regarding financial stress may be a component of the assessments, in response to the important findings of the first stage of this work regarding the contexts created through the interaction of poverty and other adversity. (2) Intervention levels and functions. The proposed intervention functions at three levels: the individual link worker and family, operational, and system/strategy levels. The intervention centres on a link worker, who develops relationships with family members, and acts as a single point of contact to understand family needs and the interaction of multiple complex adversities. They advocate for families, facilitate and coordinate system navigation, and tailor access to services. Additionally, they work at and feed into the operational level to remove barriers to services for all families at risk of or experiencing adversity. The intervention will function at the system and strategy level, through local co-creation and multi-sectoral collaboration to support system integration. (3) Foundational elements. Workforce: Rather than create new services or staff roles, the link worker role should be an element of existing services; health visiting or midwifery services may be able to include the functions of a link worker. Governance and financing: Alliance contracting would draw commitments across relevant services to work together to support the intervention.

The final stage aimed to converge on a defined intervention (Fig. 4 and Box 1) for delivery and testing, further addressing context, intervention refinement, and uncertainty as outlined in the MRC complex interventions development framework.

**Box 1:** Expected outputs and outcomes

• System-level changes
○ Prevention focus
○ Proportionate universal response
○ Multi-sectoral working, improved system integration, reduced service silos
○ Collaboration, upskilling, and professional satisfaction of service providers
○ Reduced barriers to access, improve system responsiveness
○ System efficiency
• Individual-level outputs
○ Parental support for access to services to prevent or reduce substance use, domestic violence, or mental ill health, including anti-poverty/income maximization, thereby preventing/reducing childhood exposure to ACEs
○ Support for children to access support for social and emotional needs, reducing susceptibility to the impact of exposure to ACEs
• Individual-level outcomes
○ Maximise family income
○ Parental wellbeing
○ Children’s wellbeing including reduction of socio-emotional problems, better relationships with families, more time spent at school, and increased social and educational opportunities
○ Life course impacts of ACEs, and therefore of prevention

#### Context

##### Inclusion and Service Entry Points

Policy Lab participants proposed that the optimum period for a village-style intervention may be from the perinatal period to age five, given the importance of early childhood development. (Adolescence was also discussed as a key point for entry, due to heightened risks for this age group. The decision to focus on early years was pragmatic, to simplify the intervention at this stage.) To facilitate access, remove barriers, and ensure a preventative approach (key elements recognised in the previous stages), the participants recommended that identification of children and families who would benefit from the intervention could be achieved through universal routine assessments. These assessments could take place where there are already ‘universal’ touch points in place, and in ‘critical moments’ in which children and families experience significant service or educational transitions, for example at routine perinatal appointments, through health visiting at the one-year assessment or the two-year development review, or when starting preschool or primary school. Other opportunities could be on arrival in the UK from another country, during parental separation or divorce, or following a family bereavement. Participants further suggested that a self-referral route for families and children should also be available, with touch points such as community child activity groups and schools offering signposting to support.

##### Foundational Elements: Multi-sector Working Through Link Workers in Existing Services and an Alliance Contract

Participants recommended integrating the link worker function into existing roles, such as health visitors or midwives, as avoiding the creation of new posts should reduce costs and ensure a trusted individual is embedded within local services. However, they also noted that this would be a significant change to job specifications, and may be met with resistance and the need for additional investment to extend staff capacity.

To deliver the intervention, Policy Lab participants suggested that local stakeholders implement an ‘alliance contract’, which would draw commitments across relevant services to work together to support the intervention. The agreement would include development, funding, delivery, and evaluation. Partners would work together to develop a vision statement—for example, ‘every need, met at home’- and indicate the kinds of services required for their community. The alliance would require an instigator, and commitments from other senior leaders across relevant health and local public services to build programme support.

#### Intervention Refinement

Policy Lab participants insisted that for every local implementation, families and providers must be meaningfully involved in the specific design and implementation plan of the intervention. Through local co-creation events, community members could define the required elements of their ‘village’, ensuring the intervention is tailored to community needs.

#### Uncertainty

The Policy Lab concluded that feasibility studies would be necessary to determine the acceptability of village-style interventions, and the elements necessary for scale-up. The participants also noted that it would be valuable to develop an economic case locally, to demonstrate the costs and benefits that would accrue over a defined period. Policy Lab participants recommended that local co-design would need to explore which professionals might best take the role of link worker, in perinatal or other contexts. Finally, the participants agreed that ongoing evaluations should measure outcomes for children, families and services, including an economic evaluation.

## Discussion

The ORACLE project set out to co-design a complex intervention to improve outcomes for children and young people experiencing or at risk of ACEs, through prevention and improved support. Through the mixed methods research reported here, we designed a village-style intervention within the frameworks of the Double Diamond and MRC complex interventions development guidance (Fig. 1). This research addresses two evidence gaps: first, the need for novel integrated interventions for children and families experiencing multiple adversities; and second, the need for development and testing of co-design methods for knowledge translation, describing how research evidence, professional knowledge, and lived experience can inform design, particularly for families experiencing multiple adversity.

In addressing the first research gap, the primary theoretical model for the intervention development was the concept of syndemics, in which multiple adversities interact and amplify their burden, in the context of harmful social conditions such as poverty (Tsai et al., 2017). The quantitative and qualitative work in Stage 1 highlighted the clustering of adversity, and the negative outcomes exacerbated by experience of multiple adversity in the context of poverty. This led to the focus on an integrated intervention which could address multiple adversities, and explicitly address poverty as a family exposure and intervention context, as interventions to address specific childhood adversities may not be meaningful if socioeconomic conditions are ignored. The proposed intervention aims to address poverty, by using universal assessment to promote equitable uptake, and through link workers who may support poverty proofing through income maximisation and navigating or removing barriers to access. These approaches may need to go alongside action on the wider determinants of exposure to adversity, including policies that reduce child poverty. The intervention developed in this work builds on the literature on link workers or community health workers, and social prescribing, policies in which there is significant interest but limited and low quality evidence for effectiveness and cost-effectiveness (Bickerdike et al., 2017; Kiely et al., 2022). The intervention levels and functions (see Fig. 4) are adapted from the It Takes a Village intervention (Kuchemann et al., 2021). The service entry principles and foundational elements were developed separately.

This work extends existing models of social prescribing or link worker interventions. First, the ORACLE village-style intervention focuses on prevention or early intervention, identifying families through proactive outreach and universal assessment. The included population would be those experiencing multiple adversities and financial difficulty. The link worker would then act as a single point of contact for a sustained period of months or years, allowing trusting relationships to develop gradually, and the link worker to act as a family advocate, service coordinator, and system navigator, informing about and connecting families with appropriate services. In health economic terms, the link worker is intended to improve the efficiency of a failing part of the market through reducing the asymmetry of information. We hypothesize that not only might the overall cost of services for families facing multiple adversities be reduced by taking a preventative/early intervention approach, but that connecting families with appropriate services at an early stage could provide additional health and economic benefits in the short and long-term, all of which could lead to a positive return on investment for the proposed village-style intervention. To evaluate this, it will be essential to consider different perspectives in an economic analysis, assessing the costs and benefits not only to the health and social sectors, but also for families. Crucially, the link worker should also influence the operational and strategic levels through identifying and reducing barriers to accessing services, thereby reshaping system access for all, not only for the families they support directly. A robust evaluation will be required to strengthen the evidence base for village-style interventions.

Regarding the second research gap, around methods for knowledge translation through co-design, failure to translate research into practice and policy is well documented, resulting in suboptimal and inefficient care and services. There is significant literature and guidance on knowledge translation (Graham et al., 2006; Greenhalgh et al., 2016; Grimshaw et al., 2012). However, there are few examples of intervention co-design for families experiencing multiple adversity. Recently, an integrated health and social care hub was co-designed with families experiencing adversity (Hall et al., 2023). However, this differs significantly from our work as it began with the aim of co-designing a hub, whereas the intervention type was not defined at the initiation of the ORACLE project. The lack of reported exploratory intervention design work may be due to the inherent tensions between design processes, which embrace exploration and ambiguity, and research cultures and project funding which aim to minimize risk by requiring detailed pre-specified research plans. These tensions are a challenge to authentic co-design processes (Hall et al., 2023).

Unusually, in this work we were able to take an exploratory, iterative approach, with minimal constraints on the final intervention design. This had the benefit of allowing authentic co-design processes, finding solutions to adapt services to family needs, with the active participation of stakeholders. While narrowing the focus to a single intervention was challenging, this work demonstrates that exploratory intervention design, beginning without a pre-conceived intervention type, creates opportunities for authentic co-design.

The methodological strengths of this work include rigorous methodologies, which provided a sophisticated understanding of the problem, context, and evidence gaps. This was enhanced by a systematic approach, using the MRC complex interventions core elements framework, and extensive and continuous stakeholder involvement. The resulting intervention integrates the learning across all the stages.

We expect the combination of the Double Diamond process and MRC complex intervention framework may provide a useful model for future intervention development. Both the Double Diamond process and the MRC framework have pros and cons. The concept of iterative phases of expansion and focus in the Double Diamond process was helpful for this work. However, some work may benefit from more than two rounds of this iteration, and so it should be used flexibly. We expect that further rounds of expansion and focus will be beneficial as the intervention is refined with local stakeholders ahead of and during implementation. The MRC framework provides a valuable systematic comprehensive framework of core elements. It is not prescriptive regarding steps to address each of the core elements, which allows for flexibility, but also poses challenges in the mechanics of intervention development. We hope that the example of this study may provide an adaptable template, as illustrated in Fig. 1, for future intervention co-design work.

Regarding methodological limitations, alongside the aim to be rigorous and systematic in our approach to intervention development and design, we identified points where the research team had to make decisions on intervention elements with imperfect or incomplete information. Furthermore, this research was conducted in developed, urban settings, and may not be generalizable to other settings. We also acknowledge that intervention development is complex and non-linear, and therefore difficult to capture precisely in a written manuscript.

Additionally, while we aimed to develop an intervention that would meet all of the needs and design principles identified and developed through this work, the intervention outlined nevertheless has limitations. With a focus on prevention and early intervention, we have designed a proactive intervention that could identify families in need of support through enquiry at universal service touch points. However, we recognize that there will be families who are not engaged with these services. Outreach to these families and adaption of the services offered may be needed to maximise reach. Furthermore, the described intervention does not directly include an offer specifically for young people, and this may need separate development. In addition, intervention implementation will need support from policy-makers, service funders, and providers. While we present hypotheses about the return on investment, the uncertainty and the timescales of these potential returns may not be aligned with the needs of stakeholders, who may require more certain short-term returns.

In this work, we have presented the development and design of an intervention which aims to prevent adversity and improve child outcomes. The next step will be designing a feasibility study and evaluation (proposed study design outlines are included in the Supplementary Materials) to assess the effects, costs and benefits of the resulting village-style intervention over a range of time periods.

## Supplementary Information

Below is the link to the electronic supplementary material.

Supplementary file1 (PDF 387 kb)
