# Supplementary material for: OveRcoming Adverse ChiLdhood Experiences (ORACLE): A Mixed Methods Intervention Co-design Study to Improve Outcomes for Children and Young People Experiencing or at Risk of Adversity
Source: J Prev (2022). 2025 Aug 11;47(1):1–21. doi: 10.1007/s10935-025-00866-7 (PMC12858621; doi:10.1007/s10935-025-00866-7)
Supplement: Supplementary file 1 — Supplementary file1 (PDF 387 kb) [file 10935_2025_866_MOESM1_ESM.pdf]

Supplementary Materials

Health Economics Scoping Review Results

The health economics scoping review (search conducted 9 Nov 2023) resulted in 9 peer-reviewed papers and 1 report by a private consultancy being included in the review. These included two systematic reviews (Hughes et al., 2021; Peterson et al., 2023), and 8 of which had empirical evidence on the cost of ACEs in the US (Burwick & Zaveri, 2014; Koball et al., 2019; Okwori et al., 2022; Schickedanz et al., 2019), the UK (Copello et al., 2010; Hughes et al., 2020), the Netherlands (Dijk & Kiernan, 2023) and Australia (Loxton et al., 2019). No additional papers assessed the cost-effectiveness of any interventions, but the search did identify protocols for feasibility studies and randomised trials that intended to provide an assessment of the cost-effectiveness of interventions seeking to improve childhood or adult outcomes due to adverse experiences in childhood.

The intervention costs for the provision of care and support around substance use provided by family members described in the main text (Results – Stage 1) include day-to-day costs, drug incidental costs, cost of crime on carer, and healthcare costs, as described in Table 3 by Copello et al. (2010). The estimated annual income lost by the carer of the drug using relative was also extracted from Table 3.

Burwick, A., & Zaveri, H. (2014). *Costs of early childhood home visiting: An analysis of programs implemented in the supporting evidence-based home visiting to prevent child maltreatment initiative*.

Copello, A., Templeton, L., & Powell, J. (2010). The impact of addiction on the family: Estimates of prevalence and costs. *Drugs: Education, Prevention & Policy*, *17*(Suppl 1), 63-74. https://doi.org/10.3109/09687637.2010.514798

Dijk, H. H., & Kiernan, C. K. (2023). [Societals costs of trauma]. *Tijdschr Psychiatr*, *65*(9), 563-567. (Maatschappelijke kosten van trauma.)

Hughes, K., Ford, K., Bellis, M. A., Glendinning, F., Harrison, E., & Passmore, J. (2021). Health and financial costs of adverse childhood experiences in 28 European countries: a systematic review and meta-analysis. *Lancet Public Health*, *6*(11), e848-e857. https://doi.org/10.1016/s2468-2667(21)00232-2

Hughes, K., Ford, K., Kadel, R., Sharp, C. A., & Bellis, M. A. (2020). Health and financial burden of adverse childhood experiences in England and Wales: a combined primary data study of five surveys. *BMJ Open*, *10*(6), e036374. https://doi.org/10.1136/bmjopen-2019-036374

Koball, A. M., Rasmussen, C., Olson-Dorff, D., Klevan, J., Ramirez, L., & Domoff, S. E. (2019). The relationship between adverse childhood experiences, healthcare utilization, cost of care and medical comorbidities. *Child Abuse Negl*, *90*, 120-126. https://doi.org/10.1016/j.chiabu.2019.01.021

Kuchemann, N., Akoto, E., & Southwark Health and Wellbeing Board. (2021). *Joint Southwark Mental Health and Wellbeing Strategy 2021 - 2024* https://moderngov.southwark.gov.uk/documents/s102469/Appendix%201%20-

%20Southwark%20Joint%20Mental%20Health%20and%20Wellbeing%20Strategy%202021%20-%202024.pdf
Loxton, D., Townsend, N., Dolja-Gore, X., Forder, P., & Coles, J. (2019). Adverse Childhood Experiences and Healthcare Costs in Adult Life. *J Child Sex Abus*, *28*(5), 511-525. https://doi.org/10.1080/10538712.2018.1523814
Okwori, G., Stewart, S., Quinn, M., & Lawson, D. (2022). Health Care Burden and Expenditure Associated with Adverse Childhood Experiences in Tennessee and Virginia. *J Child Adolesc Trauma*, *15*(3), 727-739. https://doi.org/10.1007/s40653-021-00390-w
Peterson, C., Aslam, M. V., Rice, K. L., Gupta, N., & Kearns, M. C. (2023). Systematic Review of Per Person Violence Costs. *Am J Prev Med*. https://doi.org/10.1016/j.amepre.2023.08.009
Schickedanz, A. B., Escarce, J. J., Halfon, N., Sastry, N., & Chung, P. J. (2019). Adverse Childhood Experiences and Household Out-of-Pocket Healthcare Costs. *Am J Prev Med*, *56*(5), 698-707. https://doi.org/10.1016/j.amepre.2018.11.019

Supplementary Figure 1

Intervention levels and functions for ‘It Takes a Village’

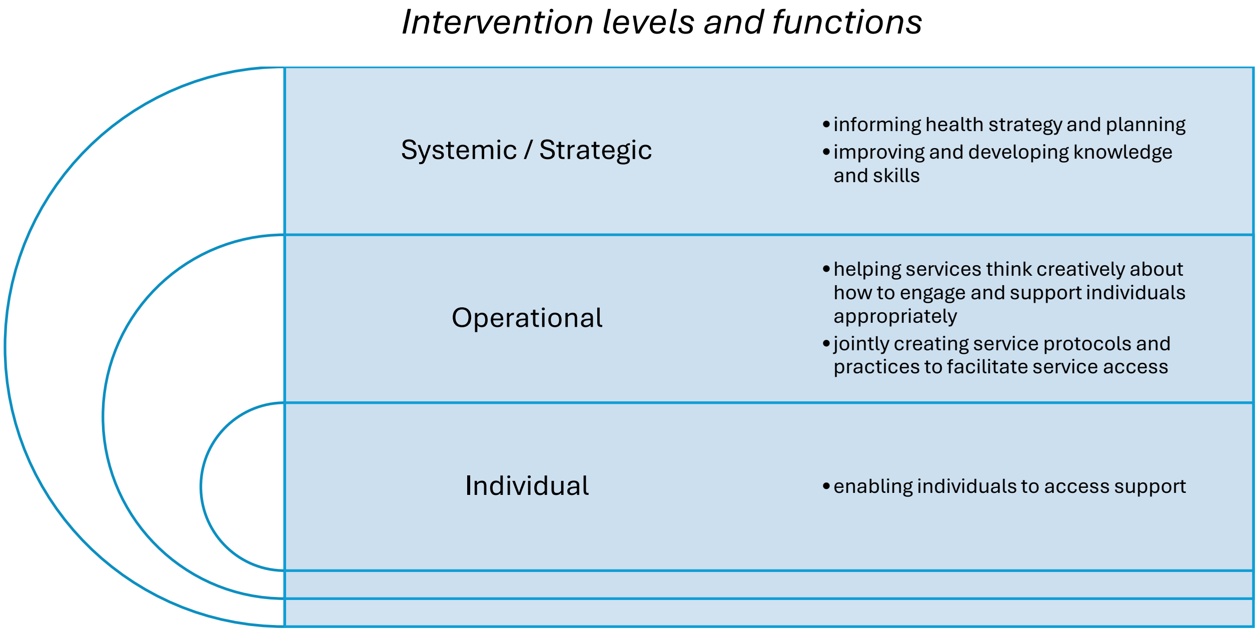

It Takes a Village responds to the challenges of people facing multiple disadvantage who find it difficult to access preventative services. It addresses these challenges on three levels, individual, operational, and systemic/strategic (Kuchemann et al., 2021).

Supplementary Table 1

Main theme	Subthemes	Intervention Context elements linked to these themes/subthemes
1. Cumulative adversity		Socioeconomic status
2. The impact of syndemic risk	2.1 Behavioural impacts	Multiple interacting risk factors
	2.2. Emotional impact and social isolation	Isolation and stigmatisation
3. Families navigating risk	3.1. Strategies to navigate risk	*not applicable to intervention context*
	3.2. Children as motivation	*not applicable to intervention context*
4. Family support	4.1. Ineffective support	Addressing complex needs
	4.2. Characteristics of good support	Key characteristics of good support

Supplementary Table 2

Summary of the study findings within each MRC Complex Intervention Framework Core Element, across the four stages.

Core Element	Stage 1	Stage 2	Stage 3	Stage 4
**Context**	• Families experience difficulties due to poverty (Interviews) and clustering of poverty and other adversities leads to worse outcomes (Quantitative analysis)<br>• Multiple adversities interact (Interviews) but most interventions address risk factors in isolation (Evidence synthesis)<br>• Interventions should be sensitive to stigma and safety (Interviews)			Policy Lab recommendations:<br>• Intervention entry through universal services and self-referral, initially focused on the perinatal period through to age five.<br>• Link-workers integrated in existing services and funded through alliance contracting
**Economic considerations**	• Costs associated with ACEs vary (Evidence synthesis)<br>• Little evidence is available on intervention costs (Evidence synthesis)		Policy Lab findings:<br>• A link-worker intervention may provide good return on investment, short and long term, by improving family outcomes and reducing system costs<br>• Investment in the intervention may be difficult without	

Core Element	Stage 1	Stage 2	Stage 3	Stage 4
			guaranteed short-term returns.	
**Uncertainty**	Uncertainty remains regarding:<br>• How poverty and family adversity interact and affect wellbeing<br>• How to provide flexibility and support multiple individuals within a family (Interviews and Evidence synthesis)<br>• Prioritisation and acceptability of approaches (Evidence synthesis)			Policy Lab recommendations:<br>• Conduct feasibility studies to assess acceptability and the requirements for scale-up.<br>• Develop an economic case locally, to demonstrate the costs and benefits.<br>• Use ongoing evaluations to measure outcomes for children, families and services, including an economic evaluation.
**Programme Theory**		• Intervention components (developed in Co-design workshops):<br>1. Connection to community resources through a relationship with a single point of contact<br>2.Trusting relationships with practitioners who understand trauma	Policy Lab findings:<br>• Link-worker may reduce barriers, understand multiple complex adversities, advocate and coordinate<br>• Expected impact/outcomes: reduction of socio-emotional problems, better relationships with families, more time	

Core Element	Stage 1	Stage 2	Stage 3	Stage 4
		3. Tailored support for each family member<br>• Practitioners can work at family, operational, and system/strategy levels (Academic workshop)	spent at school, and increased social and educational opportunities<br>• Expected staff/system outcomes: Staff satisfaction, upskilling, collaboration.	
**Intervention refinement**				• To ensure tailoring to local community needs, families and providers must be meaningfully involved in co-creating the specific design and implementation plan. (Policy Lab)
**Stakeholders**	• PPI groups provided feedback on findings and reiterated the importance of youth-specific spaces and investment in CYP mental health and wellbeing.	• Parents and young people with lived experience shared insights that informed programme theory<br>• PPI groups gave input on research planning and dissemination	• PPI groups shared their experiences of services and support<br>• Policy-lab participants endorsed a village-style approach and contributed to shaping the programme theory and economic considerations.	• Policy Lab participants refined the implementation plans, particularly around delivery context and steps for local intervention refinement, and advised on evaluation.

Supplementary Materials Text Box 1

Proposed study design outlines for Feasibility and Efficacy studies

Phase 1: Feasibility study design outline

Objectives

- Explore feasibility of delivering the ORACLE intervention, including delivery methods and barriers and facilitators to implementation
- Explore the acceptability of the intervention to parents, service providers, and wider stakeholders
- Estimate the acceptability of study design for a further efficacy study, including likely recruitment and retention of parent study participants
- Pilot and adapt methods for data collection

Settings

- Establish new ORACLE intervention services, and/or
- Work with local areas to refine existing interventions towards the ORACLE intervention

Methods

- Mixed methods, to include data collection on process and outcome measures, qualitative interviews with parents, service providers, and other key stakeholders

Outcomes and analysis

- Feasibility and acceptability will be the main outcomes, and analysis will be primarily descriptive

Phase 2: Efficacy study design outline

Objectives

- Evaluate efficacy, economics, and implementation of the ORACLE intervention

Evaluation workstreams

1. Efficacy
    a. Key outcome measures
        i. Family income
        ii. Parental wellbeing, mental health, quality of life and capabilities
        iii. Child wellbeing, mental health, quality of life and capabilities
    b. Quasi-experimental design, e.g. interrupted time series, to compare outcomes between intervention and control groups, while also evaluating in a real-world setting.
2. Efficiency (assessed alongside the quasi-experimental design for short-term impacts, with long-term impacts presented in an economic model)
    a. Adopt a societal perspective, including health and social care providers (possibly also education, criminal justice), as well as service users and their families. The service user perspective should include both the financial costs and time costs (e.g. time spent coping with mental health, substance use, domestic abuse).
    b. Assess costs of the intervention (e.g. staff time, capital and recurrent costs for set up, delivery and administration) and costs of the consequences of ACEs (e.g. health service use), with and without the intervention.
    c. Compare costs alongside i) a range a consequences in cost-consequence analysis, ii) quality-adjusted life years for cost-utility analysis and iii) monetised benefits for cost-benefit analysis.
3. Process evaluation to examine implementation, mechanism of impact, and unintended consequences
    a. mixed methods
    b. following an established framework
